# Feasibility, Safety, Enjoyment, and System Usability of Web-Based Aerobic Dance Exercise Program in Older Adults: Single-Arm Pilot Study

**DOI:** 10.2196/39898

**Published:** 2023-01-16

**Authors:** Kazuki Hyodo, Tetsuhiro Kidokoro, Daisuke Yamaguchi, Michitaka Iida, Yuya Watanabe, Aiko Ueno, Takayuki Noda, Kenji Kawahara, Sumiyo Nishida, Yuko Kai, Takashi Arao

**Affiliations:** 1 Physical Fitness Research Institute Meiji Yasuda Life Foundation of Health and Welfare Tokyo Japan; 2 Faculty of Sport Science Nippon Sport Science University Tokyo Japan; 3 Information Services International-Dentsu Ltd Tokyo Japan; 4 Meiji Yasuda Health Promotion Center Meiji Yasuda Health Development Foundation Tokyo Japan

**Keywords:** low-intensity exercise, home exercise, online exercise, supervised exercise, elderly, COVID-19, smartphone, tablet, videoconferencing platform

## Abstract

**Background:**

During the COVID-19 epidemic, opportunities for social interaction and physical activity among older people are decreasing, which may have a negative impact on their health. As a solution, a web-based group exercise program provided through a videoconferencing platform would be useful. As a web-based exercise program that older adults can easily, safely, and enjoyably perform at home, we developed a short-duration, light-intensity aerobic dance exercise program. Before studying the effectiveness of this exercise program, its characteristics, such as feasibility, safety, enjoyment, and system usability, should be examined among older adults.

**Objective:**

This pilot study aimed to examine the feasibility, safety, and enjoyment of a web-based aerobic dance exercise program and the usability of a web-based exercise delivery system using a videoconferencing platform for older adults.

**Methods:**

This study was designed as a prospective single-arm pilot study. A total of 16 older adults participated in an 8-week web-based aerobic dance program held every morning (8:30 AM to 8:50 AM) on weekdays at home. Retention and adherence rates were measured for the program’s feasibility. Safety was assessed by the heart rate reserve, an index of exercise intensity calculated from heart rate, and the number of adverse events during exercise sessions. Enjoyment of this exercise program was assessed by an 11-point Likert scale ranging from 0 (not enjoyable at all) to 10 (extremely enjoyable) obtained through telephone interviews after the first-, third-, sixth-, and eighth-week intervention. For usability, the ease of the videoconferencing platform system was assessed through telephone interviews after the intervention.

**Results:**

A female participant with hypertension dropped out in the second week because of the continuously reported high blood pressure (≥180 mmHg) before attending the exercise session in the first week. Therefore, the retention rate was 93.8% (15/16). Among the remaining participants, the median (IQR) overall adherence rate was 97.4% (94.7-100). Regarding safety, the mean (SD) heart rate reserve during the aerobic dance exercise was 29.8% (6.8%), showing that the exercise was relatively safe with very light to light intensity. There were no adverse events during the exercise session. The enjoyment score (0-10 points) significantly increased from the first (6.7 [1.7]) to sixth (8.2 [1.3]) and eighth week (8.5 [1.3]). Regarding usability, 11 participants reported difficulties at the beginning, such as basic touch panel operations and the use of unfamiliar applications; however, all got accustomed to it and subsequently reported no difficulty.

**Conclusions:**

This study showed high feasibility, enjoyment, and safety of the web-based aerobic dance exercise program in older adults, and the web-based exercise delivery system may have areas for improvement, albeit without serious problems. Our web-based aerobic dance exercise program may contribute to an increase in physical and social activities among older adults.

## Introduction

Previous studies have reported that physical activity and social interaction positively influence the physical, cognitive, and mental health of older adults [1-4]. However, for some, access to places for exercise and social participation is difficult due to environmental limitations (such as limited transportation, low walkability, and remote location) as well as physical limitations and social factors [5,6]. During the initial period of COVID-19, the existing barriers were exacerbated because of political countermeasures to stay home and the closure of public sports and recreational facilities [7,8]. Although several restrictions have been eased, COVID-19 is ongoing, and some older people have not restarted physical activity [8]. Therefore, opportunities to participate in physical and social activities at home are crucial.

To address these issues, a solution could be a web-based exercise program at home [9,10]. With the development of communication technology, people can participate in group exercises at home with guidance from an instructor through videoconferencing platforms such as the Zoom application (Zoom Video Communications) [11,12]. Although a web-based exercise through videoconferencing platform is beneficial, the feasibility, safety, and enjoyment of the web-based exercise program and ease of use of a web-based exercise delivery system should be carefully considered especially in older adults [13]. Feasibility can be assessed using the individuals’ retention and adherence rates. As a program for older adults, safety is important because participants exercise at home without receiving on-site support from an instructor or supervisor. To mitigate the risk of adverse events during the web-based exercise program, light intensity and short duration of exercises are preferred. Additionally, to ensure its wide acceptance and long-term use, it should be easy to implement and enjoyable [14]. Regarding the system’s usability, its operations should be simple and easy for older adults because technical issues are the main barriers to using information and communication technology (ICT) devices [9,15].

Then, we have focused on a short-duration (20 minutes) and light-intensity aerobic dance exercise program considering these conditions. Dance is widely accepted as a group exercise for older adults [16] and does not need equipment. To safely perform dance exercises in a confined space at home, we developed a light-intensity aerobic dance program mainly comprising upper limb and trunk movements and confirmed that it was enjoyable for the older adults to perform, and it transiently enhanced cognitive function and mood in a laboratory setting [17,18]. Moreover, to deliver the web-based exercise program, we focused on the Zoom videoconferencing application on a tablet device because it is free and widely used in society, and recent studies reported no major problems with its usability among older adults [13,19,20]. Although several studies have examined the feasibility and effectiveness of web-based exercise programs using strength training, aerobic exercise, and yoga [13,19,20], to the best of our knowledge, no prior study has investigated this kind of short-duration and light-intensity web-based aerobic dance exercise program among older adults.

Therefore, before assessing its effectiveness, as a pilot study, we conducted a single-arm intervention to examine the feasibility, safety, and enjoyment of the web-based aerobic dance exercise program, alongside the usability of the exercise delivery system among older adults.

## Methods

### Participants

The participants were recruited from community-dwelling older adults who belonged to the Hachioji medical consumer cooperative. This organization provides medical and nursing support during illness or frailty, and members range from those in good health to those with some form of illness. The staff of the cooperative distributed flyers about the study to community members, and 24 interested older adults (n=6, 25% male and n=18, 75% female) voluntarily participated in an information session where they were fully informed about its purpose and experimental procedures. After the session, 8 participants declined to participate (7 did not match the schedule and 1 was unable to exercise due to sciatica). Finally, 16 older adults (n=4, 25% male and n=12, 75% female) provided their written informed consent. The inclusion criteria were as follows: aged 65 years or older; living in Hachioji City, Tokyo; no restriction regarding exercise by a medical doctor because of a cardiovascular or orthopedic disease; and no diagnosis of mental illness, including dementia. Given this study’s exploratory nature, no power analysis was performed to determine the sample size. Table 1 shows the participants’ demographic data.

**Table 1:** Characteristics of the participants (N=16).

Variable		Value
Age (years), mean (SD)		77.6 (4.5)
**Sex, n (%)**		
	Male	4 (25)
	Female	12 (75)
Living alone, n (%)		7 (43.8)
Current working, n (%)		2 (12.5)
**Social participation^a^, n (%)**		
	No	4 (25)
	Yes	12 (75)
**Physical activity or exercise^a^, n (%)**		
	No	1 (6.3)
	Yes	15 (93.8)
**Clinical treatment^b^, n (%)**		
	No	6 (37.5)
	Yes	10 (62.5)
**Pain status^c^, n (%)**		
	No	5 (31.3)
	Yes	11 (68.8)
**Devices ownership, n (%)**		
	PC	6 (37.5)
	Cell phone	4 (25)
	Smartphone	13 (81.3)
	Tablet	3 (18.8)
**Purpose of internet use, n (%)**		
	Email	7 (43.8)
	Messaging app	10 (62.5)
	Information collection	12 (75)
	Videoconference	1 (6.3)
	Shopping	2 (12.5)
	Online game	1 (6.3)
	Audios or videos	8 (50)

^a^Classified as “yes” if participating at least once a month.

^b^Classified as “yes” if receiving any treatment.

^c^Classified as “yes” if feeling any physical pain.

### Ethics Approval

The ethics committee of the Physical Fitness Research Institute of Meiji Yasuda Life Foundation of Health and Welfare approved the study protocol (approval number: 2020-0001).

### Procedures

The participants took part in an 8-week web-based aerobic dance exercise program through the videoconferencing platform using a tablet device at home. Before starting the intervention, we lent 4 electronic devices to every participant free of charge for accessing the exercise program and monitoring their heart rate (HR) during exercise, as follows: (1) tablet (iPad [Apple Inc]) for web-based exercise, (2) HR monitoring device (OH1 [Polar Electro Oy]), (3) smartphone (iPhone SE2 [Apple Inc]) for HR measurement, and (4) Wi-Fi router (Pocket WiFi 801ZT [Softbank]) with a maximum downstream speed of 112.5 Mbps and a maximum upstream speed of 37.5 Mbps for data transmission. As the recommended bandwidth for group video calling on Zoom is 3.8 Mbps/3.0 Mbps (up/down), we prepared the Wi-Fi router that met these requirements. We paid for the data communication fees used in this study. Before lending these devices, we installed LINE WORKS (WORKS MOBILE Corp) and Zoom on the iPad and the HR monitoring app on the iPhone for each participant. LINE WORKS is a messaging app that includes group chat, calling, and survey functions. We used it to send the participants the Zoom URL and check their health condition through a simple checklist on the app.

**Figure 1:**
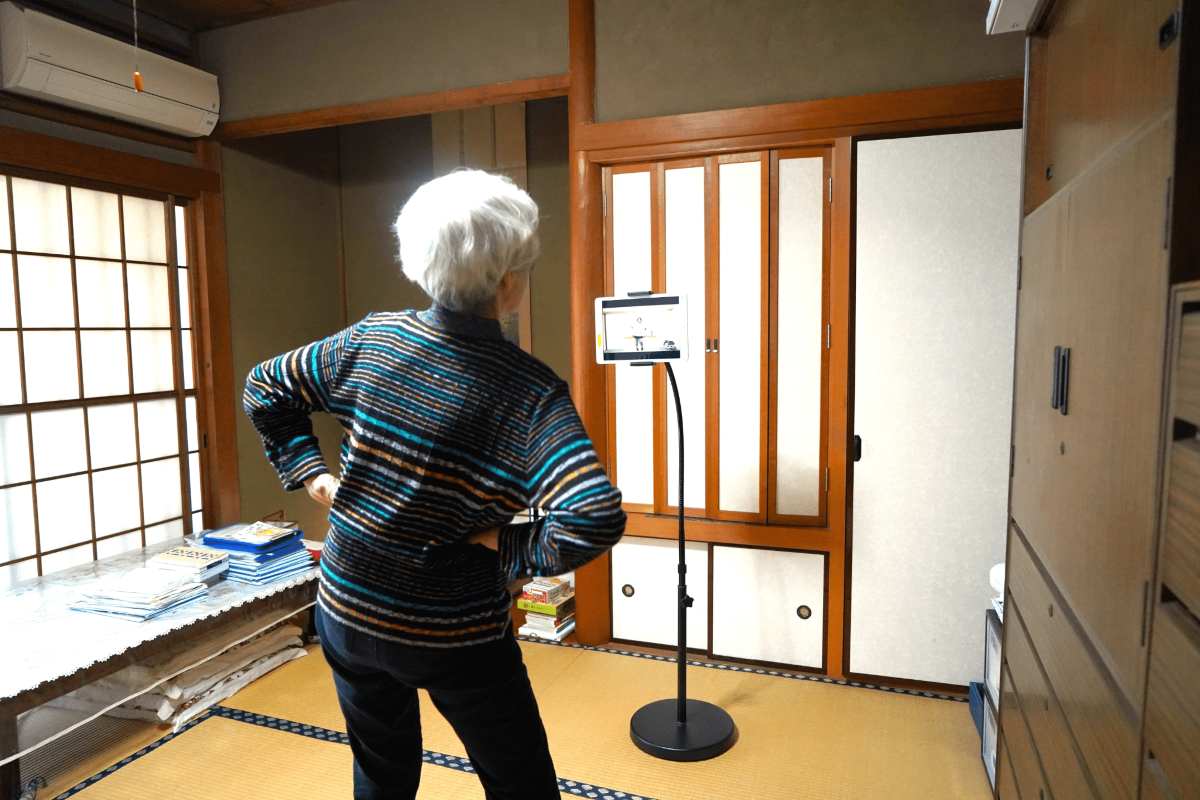
Participant exercising at home. Participants exercised following the guidance of the instructor via tablet. All the devices were set up by the research staff.

During the exercise session, the HR of each participant was measured with the monitoring device and tracked on the screen at the laboratory in real time by the research staff. Moreover, participants’ subjective feelings regarding enjoyment of the exercise program were obtained through telephone interviews at the end of the first, third, sixth, and eighth weeks of the intervention. Figure 1 shows the participants exercising at home using the devices.

### Exercise Program

The total duration of the web-based exercise program was 20 minutes, comprising a 5-minute warm-up (stretching), a 10-minute light-intensity aerobic dance exercise called Slow Aerobic Dance Exercise (SADE), and a 5-minute cooldown (stretching). The details of the SADE have been reported in a previous study [11]. Briefly, it included 3 upper-body dynamic movements (twisting the upper body, pulling back the elbows, and clapping hands while shaking the waist from side to side, and waving arms like wiping the windows while shaking the waist from side to side). All movements were performed to music with a tempo of 90-120 beats per minute (bpm) because we confirmed that older adults can comfortably practice the aerobic dance program at 90-120 bpm [17,18]. Additionally, the movements were slightly changed every 2 weeks to avoid boredom. Moreover, 1 of the 3 female professional aerobic exercise instructors led the exercise program using Zoom. The exercise started at 8:30 AM, from Monday to Friday, for 8 weeks without national holidays (February 1 to March 27, 2021). In total, 37 sessions were conducted during the intervention period.

### Web-Based Exercise Delivery System

We used Zoom as the videoconferencing platform to deliver the web-based exercise program to the participants. On the morning of the exercise day, the Zoom URL was sent to the participants through LINE WORKS. The participants only needed to access the LINE WORKS application and click on the Zoom URL to participate in the web-based exercise session.

### Safety Management

To monitor and manage participants’ safety during the exercise, we obtained information on their health conditions using a simple checklist on LINE WORKS every morning before the session. Moreover, we monitored their movements and HR during the exercise. The simple health checklist included the following three questions: (1) “How is your overall health condition today?” (Excellent/Good/Poor), (2) “What is your systolic (SBP) and diastolic (DBP) blood pressure before the session?” (SBP: less than 140 mmHg/140-179 mmHg/180 mmHg or higher; DBP: less than 90 mmHg/90-109 mmHg/110 mmHg or higher), and (3) “Did you attend the session today?” (yes or no). Every participant had to measure their resting blood pressure before starting the session at home using an automatic sphygmomanometer provided by the research team. Before the session, the research staff members checked their answers. Regarding the overall health condition, if any participant answered “Poor” on the day of the session, a research staff called them to check how they were feeling and whether they could safely participate. Regarding blood pressure, based on the report of the Ministry of Health, Labor, and Welfare [21], an individual with an SBP of 180 mmHg or higher or DBP of 110 mmHg or higher was suggested not to participate in the exercise on the day for safety reasons. Additionally, in case of adverse events (eg, falls, injuries, or cardiovascular events) during the intervention, we were prepared to call the participant, a relative living with the participant, or someone who lived nearby to check on their situation and to call an ambulance in case of an emergency.

An overview of the web-based HR monitoring system is shown in Multimedia Appendix 1. Every participant was asked to wear the HR monitor on their upper arm before the exercise began. Each participant’s HR was displayed on the iPhone app through Bluetooth, and the HR information was transmitted to a web application on a PC at the laboratory using the internet in real time. Using this system, we monitored the HR and heart rate reserve (HRR) of each participant during the exercise. Before starting the session, we checked if the HR was displayed correctly on the web application. If someone’s HR was not displayed due to Bluetooth, Wi-Fi, or other issues, the participant called the research staff, or we called them to solve the problem. The HRR of each participant was calculated using the following equation:

HRR (%)=(HR during exercise – resting HR) / (predicted maximum HR – resting HR) × 100

**Figure 2:**
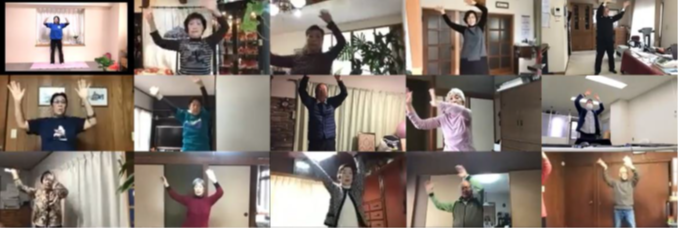
All participants shown on a monitor screen in the laboratory. The research staff checked the movements of the participants on the screen for safety during the exercise session.

The predicted maximum HR was calculated using the following equation [22]: 208 – 0.7 × age. The resting HR was measured during a visit to the participants’ homes to install apps and set up the devices. Each participant managed to exercise at an intensity of less than 60% of their HRR throughout the exercise because exercise above 60% HRR is defined as high intensity according to the American College of Sports Medicine [23], and the risk of cardiovascular events may be higher [24]. If the HRR of the participant remained over 60% for a few minutes, the research staff called them to confirm whether the exercise intensity was appropriate, advising them to slow down the exercise movement according to the situation. Along with monitoring the HR, we checked whether the participants were exercising safely and correctly. Figure 2 shows the monitoring screens of each participant’s movement during the session.

### Measures

#### Feasibility

The retention rate was calculated as the proportion (expressed as a percentage) of the number of participants who completed the 8-week assessment to the total number. The adherence rate was calculated individually as the percentage of the sessions attended to the total number (37 sessions) throughout the intervention period, and the average adherence rate was obtained.

#### Safety

Safety was examined in terms of the intensity of the exercise program and the number of adverse events. The exercise intensity was measured using the average HR and HRR during SADE across all sessions for each participant. An adverse event was defined as any unfavorable health-related events such as falls, injuries, or cardiovascular events that occurred during the exercise intervention.

#### Enjoyment

Regarding enjoyment, the participants were asked to rate their subjective enjoyment on a scale at the end of weeks 1, 3, 6, and 8 of the intervention during telephone interviews. Enjoyment was assessed by a single question, “How do you feel about the exercises?”. Participants were asked to rate them on an 11-point Likert scale ranging from 0 (not enjoyable at all) to 10 (extremely enjoyable).

#### Usability of the Web-Based Delivery System

The usability of the system was evaluated from the participants’ perceived experiences with using the devices providing a web-based exercise program and monitoring HR (tablet, OH1, smartphone, and Wi-Fi router). They were asked to provide their perceived experiences using the devices over the phone by research staff. Telephone interviews were conducted after the last session of the intervention using a single question, *“*What do you think about your experiences with using the system for delivering web-based exercises?*”*. For the usability assessment, the interviewer recorded the participants’ comments and extracted the main ones. The participants were classified into the following 3 categories owing to their comments based on the discussions among all the coauthors: (1) category A: those who reported no problems with using the system throughout the intervention period; (2) category B: those who reported some challenges at the beginning but gradually got used to it or could use it without support by the end of the intervention; and (3) category C: those who reported difficulty or unfamiliarity throughout the intervention period.

### Statistical Analysis

The participants’ adherence rates to the session, as well as HR and HRR during the exercise, were reported as mean (SD) or median (IQR) whenever they comprised either a normal or nonnormal distribution, respectively. To obtain the enjoyment score, a repeated measure ANOVA was conducted to examine the change in the enjoyment of the exercise program during the intervention period (weeks 1, 3, 6, and 8). If a significant difference was observed in the ANOVA, the post hoc multiple comparisons with Bonferroni correction were performed. For the assessment of the system’s usability, 3 categories were tested using the chi-square test for goodness of fit. Furthermore, Cohen *d* effect size was calculated to assess the change in outcome measure in response to the intervention, while *ηp^2^* was computed as a measurement of the effect size for ANOVA. A significant level was set at 0.05 for all analyses. We employed R version 4.1 (R Foundation for Statistical Computing) [25] for the data analysis.

## Results

### Feasibility: Retention and Adherence

One female participant with hypertension continuously reported high SBP (≥180 mmHg) before attending the exercise since the first session. Based on the safety concerns, in the second week, we suggested that she consult a family doctor and withdraw from this study. Therefore, 15 participants completed the 8-week intervention, resulting in a retention rate of 93.8%. For the analysis of adherence, data from the remaining participants (n=4, 27% male and n=11, 73% female) were used. The median (IQR) adherence rate was 97.4% (94.7-100).

### Safety

Based on 15 participants, the mean (SD) overall HR and HRR during the SADE were 93.4 (5.7) bpm and 29.8% (6.8%), respectively, indicating the SADE was light intensity for the participants [26]. No adverse event was reported during the exercise session throughout the entire intervention period.

### Enjoyment

The mean (SD) enjoyment scores of the exercise program at weeks 1, 3, 6, and 8 were 6.7 (1.7), 7.5 (1.4), 8.2 (1.3), and 8.5 (1.3), respectively. The repeated measures ANOVA revealed a significant difference among the weeks—*F*_1.98, 27.73_=7.67, *P*=.002, *ηp^2^*=0.35. Post hoc analysis with Bonferroni correction showed that enjoyment scores at weeks 6 and 8 were significantly higher than at week 1—*t*_14_=–3.14, *P*=.04, *d*=–0.81; *t*_14_=–3.81, *P*=.01, *d*=–0.98, respectively).

### System Usability

The results from the telephone interview to assess the system usability of the web-based exercise showed that 4 (27%) participants had no problems (category A), 11 (73%) had some challenges at the beginning but got used to it or were able to use it without support at the end of the intervention (category B), and none reported any difficulties or unfamiliarity in use throughout the entire intervention period (category C). The chi-square test for goodness of fit showed significant differences between category B and category A or C.

## Discussion

### Principal Findings

A single-arm pilot study was conducted to evaluate the feasibility, enjoyment, and safety of the web-based aerobic dance exercise program and the usability of the web-based system for providing the exercise program for older adults. In this study, the following findings were obtained: (1) regarding feasibility, the retention rate and average adherence rate were high; (2) regarding safety, the average HRR during the SADE was within the range of light-intensity exercise, and there were no adverse events during the sessions; (3) regarding enjoyment, the score for the exercise program increased gradually until the end of the intervention period; and (4) regarding system usability, about a third of the participants faced some challenges in using this system at the beginning, but all got to use it at the end. These results suggest that our web-based aerobic dance program is highly feasible, enjoyable, and safe for older adults, with some areas that could be improved in the web-based exercise delivery system.

### Comparison With Previous Research

A recent systematic review based on 22 selected papers reported the process characteristics of technology-based exercise intervention programs [27]. It highlighted that there were marked differences in the attrition (0%-36%) and adherence rates (67.78%-100%) for a technology-based group training exercise program for older adults. The authors suggested that the differences in these variables were because of the varying intervention characteristics, such as implementation time; frequency; duration; intensity; mode of exercise program, including interactive or noninteractive communication, individual or group, with or without a supervisor; technology-based delivery system component, such as commercially available or customized technology systems; and participants’ characteristics, such as living independently or institutionalized. In this study, the participants showed high retention rates (15/16, 93.8% participants) and adherence rates (36/37, 97.4% sessions) to the web-based exercise program, which is consistent with the results of a previous study that used the similar intervention characteristics, such as intervention period, sample age, living status, home setting, and session duration. Schoene et al [28] reported a retention rate of 83.3% (15/18) and an adherence rate of 100% for an unsupervised, home-based, 15- to 20-minute dance step exercise program using a video game. Daly et al [29] suggested that an exercise program was feasible if the retention rate was higher than 90% and the average overall adherence rate was higher than 75%. Considering this, our exercise program was highly feasible for older adults. This seems to be related to the characteristics of the exercise, including an aerobic dance with light intensity and short session time. Dance exercise is widely accepted as a group exercise for older adults in Western countries [16]. This exercise type also seems to be acceptable for Japanese older adults. Moreover, even though the frequency (5 days/week) of our exercise program was higher than that (3 days/week) in the study by Schoene et al [28], the retention rate was a little higher. This might be because the participants were supervised using a videoconferencing platform during the session by the instructor, and they conducted the session at the same time every morning on weekdays. Kim et al [30] reported high feasibility (100% in retention and adherence rates) of their exercise intervention using Tai Chi and yoga programs, with 60 minutes per session, 3 days per week, for 8 weeks. However, they conducted the intervention using a motion capture sensor and virtual reality avatar to provide real-time visual feedback of the movement at the university research laboratory, which would not be practical as a home-based intervention program.

In a previous systematic review, Valenzuela et al [27] showed that the reasons for dropouts from their technology-based exercise intervention included low motivation, loss of interest, arthritic discomfort, lack of time, inability to travel to the session, limited space at home to set up the system, inability to use the technology, and being ashamed of playing computer games. Considering these reasons, the high feasibility of our exercise program might be because of the high level of enjoyment and safety. The participants showed a relatively high enjoyment score at the end of the first intervention week, and thereafter, there was a gradual increase during the intervention period. These results showed that the exercise program was enjoyable for the participants throughout the period. This seems to be partly because of the adaptation of the exercise program to each participant’s skill and ability. This might have reduced their state of boredom and increased their motivation. Additionally, our exercise program was constructed with light intensity and simple movements, which are favorable for older adults [31]. These conditions might have motivated the participants to engage in the exercise session every morning throughout the intervention period. This was also supported by the participants’ feedback through telephone interview after the intervention. Many participants commented that the instructors were very professional and friendly, making it easy to follow the aerobic dance program. Furthermore, they reported that the movements of the aerobic dance were not too difficult, and they could catch up even if they skipped or missed the sessions. They also experienced feeling refreshed after the exercise, which made them want to continue the program.

Regarding safety, we examined exercise intensity during the SADE using the HRR and the number of adverse events. The average HR and HRR during the exercise had a mean of 93.4 (SD 5.7) bpm and a mean of 29.8% (SD 6.8%), respectively. The average HRR ranged from very light to light intensity according to the American College of Sports Medicine guideline [26], showing that the exercise intensity was relatively safe. Regarding adverse events, any adverse events during the exercise session, such as cardiovascular events, falls, or injuries, did not occur. Among the previous technology-based exercise intervention studies, there were a few cases of knee and calf pain during strength training [29] and a fall during an exergame intervention using a balance board [32]. Since the aerobic dance exercise in this study mainly comprised upper body movements and was not physically demanding, it is considered that the risk of falls was low and joint and muscle pain did not occur.

From a safety management perspective, it should be mentioned that 1 participant with hypertension repeatedly reported high SBP (≥180 mmHg) before attending the exercise session since the first session. Although the causal relationship between the intervention and reported high SBP is unclear, it is possible that the anxiety to participate in the web-based exercise session elicits psychological stress, and this consequently causes high SBP. This case suggests the importance of management of blood pressure before attending the web-based exercise session to reduce the risk of exercise-related adverse events.

Another important condition of a web-based exercise program for older adults is the system’s usability. We examined this condition through telephone interviews at the end of the intervention. Since nobody reported any difficulties or unfamiliarity in using the system at the end of the intervention, there could be no serious problems with its usability. This is partly consistent with the previous study reporting that the operation of Zoom for participating in web-based exercise classes was easy for older adults [13]. However, about a third of the participants had some challenges using it at the beginning, suggesting that there could be usability improvements. According to the participants’ comments, a difficult point was the touch panel operations of smartphones or tablets, such as tapping, scrolling, and swiping. Some participants also commented that when operating unfamiliar apps, such as Zoom and LINE WORKS, it was difficult to know what to do next when unfamiliar screens appeared due to unusual operations. Although many had smartphones or tablet devices, most of them had never used the lent devices or apps, and it could take them some time to get used to them. For further improvement in usability, it may be necessary to make the operation of devices easier and older-adults–friendly, such as by reducing the number of taps to participate in the web-based exercise and providing detailed instructions on how to use devices and apps [33].

Moreover, it should be considered that the high rate of smartphone or tablet ownership among participants (87%) may have contributed to ensuring no significant usability issues. According to a recent national survey on the use of communication devices, the rate of internet users was 73.4% among 60-year-old people and 40.8% among those aged ≥70 years in Japan [34]. Therefore, older adults unfamiliar with ICT may have some problems using this web-based exercise delivery system. Although the number of internet users in the older population has been rapidly increasing in Japan [35], further examination of usability among older adults with low ICT literacy is needed.

### Limitations and Future Directions

In this study, there are several limitations and, hence, further research is required. First, participants’ characteristics could influence the results. The participants were volunteers, and many belonged to the community organizations as leaders. Therefore, they could have been highly motivated to participate in the intervention program, which may have influenced their adherence rate and enjoyment. Moreover, many participants owned smartphones or tablets, which may have contributed to the results of the system’s usability. Further studies with a larger sample size are needed to examine whether similar results can be obtained with older adults who are physically and socially inactive or are unfamiliar with using ICT devices. Second, to strictly examine the safety of the exercise program, we used a smartphone and HR monitor to check the exercise intensity, and asked research staff to monitor participants’ health condition and movements during the exercise session. However, from a cost-effectiveness standpoint, the system needs to be simplified for implementation in the real world. Finally, since this is a single-arm pilot study, the effectiveness of the exercise program is unknown. A future study on the effect of this web-based exercise program on physical, mental, and cognitive function in older adults should be conducted using a randomized controlled design.

### Conclusions

This pilot study indicates that an 8-week intervention using a web-based aerobic dance program with short duration (20 minutes), high frequency (5 days per week), and light intensity (HRR 29.8%) is feasible, safe, and enjoyable for older adults. Moreover, although there were no serious issues with the web-based exercise delivery system using a videoconferencing platform, some areas for improvement were found. These results suggest that our web-based exercise program could be valuable in enabling healthy behaviors for older adults. Future studies on the generalizability of the results and effectiveness of this web-based aerobic dance exercise program and usability improvements for real-world implementation are needed.

## Appendix

Multimedia Appendix 1 Overview of the heart rate (HR) online monitoring system. Participants’ HR was measured by an OH1 HR monitor on their arm, and the data were sent to the smartphone app via Bluetooth. The participants could see their HR on the smartphone app. Moreover, the HR data were sent to the web application via the internet, and the research staff could check the HR of all participants in one screen. Once the name of a participant was clicked, the time series data on their individual HR would be displayed.

## Abbreviations

bpm: beats per minute
DBP: diastolic blood pressure
HR: heart rate
HRR: heart rate reserve
ICT: information and communication technology
SADE: Slow Aerobic Dance Exercise
SBP: systolic blood pressure

## Data Availability

The data that support the findings of this study are available from the corresponding author (KH) upon reasonable request.
