# Supplementary figures and images for: Feasibility, Safety, Enjoyment, and System Usability of Web-Based Aerobic Dance Exercise Program in Older Adults: Single-Arm Pilot Study
Source: JMIR Aging. 2023 Jan 16;6:e39898. doi: 10.2196/39898 (PMC9947869; doi:10.2196/39898)

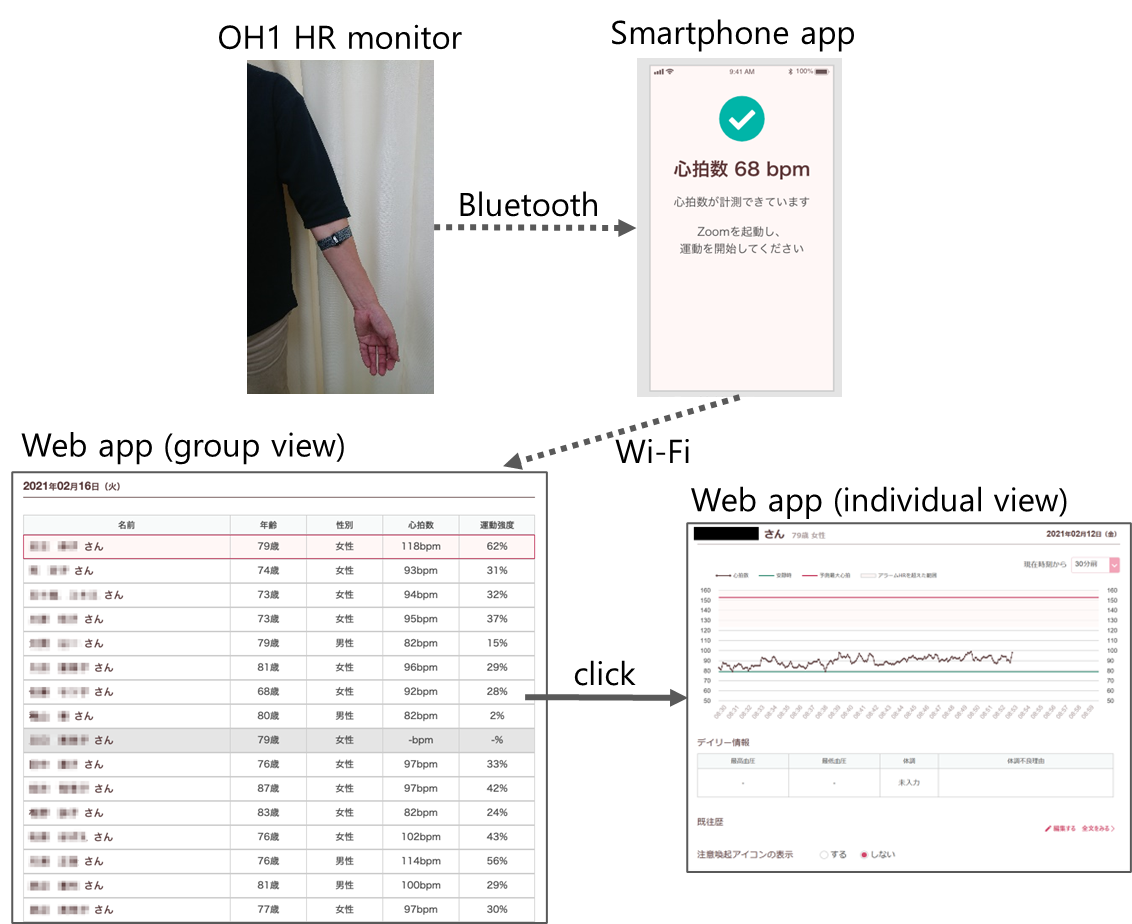

Supplement: Multimedia Appendix 1 [file aging_v6i1e39898_app1.png]
